# Software for Correcting the Dynamic Error of Force Transducers

**DOI:** 10.3390/s140712093

**Published:** 2014-07-07

**Authors:** Naoki Miyashita, Kazuhide Watanabe, Kyouhei Irisa, Hiroshi Iwashita, Ryosuke Araki, Akihiro Takita, Takao Yamaguchi, Yusaku Fujii

**Affiliations:** 1 Education Program of Electronics and Informatics, Mathematics and Physics, Graduate School of Science and Technology, Gunma University, 1-5-1 Tenjin-cho, Kiryu, Gunma 376-8515, Japan; E-Mails: t10306070@gunma-u.ac.jp (N.M.); t09306070@gunma-u.ac.jp (K.W.); t08306011@gunma-u.ac.jp (K.I.); t08306013@gunma-u.ac.jp (H.I.); t10306004@gunma-u.ac.jp (R.A.); 2 Division of Mechanical Science and Technology, School of Science and Technology, Gunma University, 1-5-1 Tenjin-cho, Kiryu, Gunma 376-8515, Japan; E-Mails: takita@el.gunma-u.ac.jp (A.T.); yamagme3@gunma-u.ac.jp (T.Y.)

**Keywords:** inertial force, dynamic force, dynamic calibration, Levitation Mass Method, error correction

## Abstract

Software which corrects the dynamic error of force transducers in impact force measurements using their own output signal has been developed. The software corrects the output waveform of the transducers using the output waveform itself, estimates its uncertainty and displays the results. In the experiment, the dynamic error of three transducers of the same model are evaluated using the Levitation Mass Method (LMM), in which the impact forces applied to the transducers are accurately determined as the inertial force of the moving part of the aerostatic linear bearing. The parameters for correcting the dynamic error are determined from the results of one set of impact measurements of one transducer. Then, the validity of the obtained parameters is evaluated using the results of the other sets of measurements of all the three transducers. The uncertainties in the uncorrected force and those in the corrected force are also estimated. If manufacturers determine the correction parameters for each model using the proposed method, and provide the software with the parameters corresponding to each model, then users can obtain the waveform corrected against dynamic error and its uncertainty. The present status and the future prospects of the developed software are discussed in this paper.

## 1. Introduction

Force transducers are widely used in both the industrial and research areas. However, at present only static force calibration methods using static standard forces are available. As a result of this fact it is very difficult for users to estimate the dynamic errors of force transducers and correct those errors.

A method, *i.e.*, the Levitation Mass Method (LMM), has been proposed for dynamic force calibration [1,2]. In the LMM, varying forces, such as an impact force, an oscillation force and a step force, are applied to the transducer as the inertial forces of a mass supported using an aerostatic linear bearing, *i.e.*, the moving part of the bearing. Then, the forces measured as the inertial force are compared with the output signal of the transducer. In the method, only the Doppler frequency shift of the laser light reflected on the mass is accurately measured using an optical interferometer. Then all the other quantities, such as velocity, acceleration, displacement and inertial force, are calculated from the frequency.

Xu *et al.* proposed a method in which the inertial mass of a part of the transducer itself is supposed to be the error source and its value is estimated from the free oscillation of the transducer after applying an impact load [3]. However, in the method, the real force during the impact is unknown and that is not used in the calibration.

Using the LMM, Fujii *et al.* experimentally showed that the error in impact force measurement is almost proportional to the acceleration at the sensing point of an S-shaped strain-gage force transducer, and further, it can be explained as the effect of the inertial force of a part of the transducer itself. The difference between the static and dynamic characteristics of the force transducer is well explained as the inertial force of a part of the transducer itself [4]. This result suggests that the dynamic measurement errors can be corrected by measuring the acceleration at the sensing point.

Based on this result, a force sensor, which consists of a strain-gage force transducer and an accelerometer, is proposed [5]. The effect of the inertial mass of a part of the sensor is corrected using the acceleration measured using the accelerometer.

Then, it is shown that the dynamic errors of force transducers can be corrected using their own output signal [6]. It is experimentally confirmed that the acceleration at the sensing point of the transducer is almost proportional to the differential coefficient of the second order of the output signal of the transducer itself. This can be understood by the fact that the deformation of the transducer is almost proportional to the force applied. However, only one transducer is used in the experiment.

In this paper, the validity of the proposed method for correcting the dynamic error and for evaluating its uncertainty is experimentally evaluated using three force transducers of the same model. Based on the experimental results, software, which corrects the dynamic error of force transducers using their own output signal, has been developed. We suggest that if the sensor manufacturers provide the software with sets of the parameters corresponding to their models, then the users can obtain the corrected waveforms and reduce the uncertainty in dynamic force measurements.

## 2. Experiment

**Figure 1.**
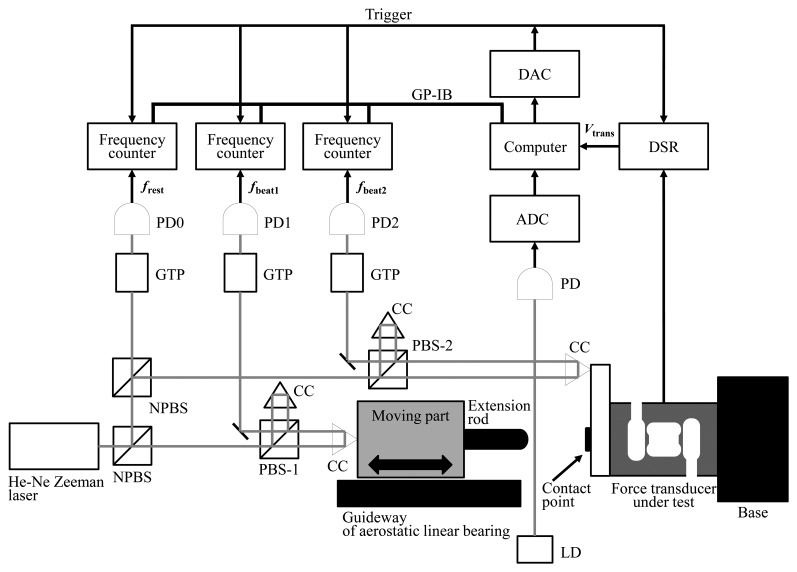
Experimental setup. Code: LD = Laser diode, PD = Photo detector, CC = Corner cube prism, PBS = Polarizing beam splitter, NPBS = Non-polarizing beam splitter, GTP = Glan-Thompson prism, ADC = Analog-to-digital converter, DAC = Digital-to-analog converter, DSR = Dynamic strain recorder.

Figure 1 shows schematic diagram of the experimental setup for measuring the impact response of force transducers. The transducer under test is fixed on a large metal base of 10^3^ kg. An aerostatic linear bearing is used in order to obtain a linear motion with sufficiently small friction acting on the mass, *i.e.*, the moving part of the bearing. The initial velocity is given to the moving part by hand.

A rubber block of sufficiently small mass is attached at the sensing point in order to adjust the steepness of the impact. The velocity of the mass *v*_1_ and the velocity of the sensing point of the transducer *v*_2_ are measured using two optical interferometers named Interferometer-1 and Interferometer-2, respectively. The force acting on the sensing point is obtained by the equation of motion, *F* = *ma*, where *m* is the mass and *a* is the acceleration.

The mass of the moving part including the cube corner prism (CC) and the extension rod, *M*_1_, is approximately 2.653 kg. The mass of the contact point of the transducer, including the CC and their base plate, *M*_2_, is approximately 0.082 kg.

Three conventional S-shaped strain-gage type transducers (model: DB-200N, capacity: 200N manufacturer: Showa Measuring Instruments Co. Ltd., Tokyo, Japan)—Transducer-1, Thransducer-2 and Transducer-3—are used in the experiment. The year of manufacture of Transducer-1 is 2000, and that of the other two transducers is 2008.

A Zeeman-type two-frequency He-Ne laser is used as the light source of the optical interferometers. The interferometers have three photo-detectors: PD0, PD1 and PD2. The frequency difference between the two orthogonal polarization states emitted from the laser, *f*_rest_, is monitored using a Glan-Thompson prism (GTP) and the first photo-detector, PD0.

The total force acting on the moving part of the aerostatic linear bearing, *F*_mass_, is calculated as the product of its mass, *M*_1_, and its acceleration, *a*_1_, as follows:

(1) $$F_{\text{mass}}=M_{1}a_{1}$$

In the measurement, the total force acting on the mass, *F*_mass_, is considered to be the same as the force acting on the mass from the force transducer being tested, since the frictional force acting on the mass is negligible [7]. The acceleration is calculated from the velocity of the levitated mass. The velocity of the mass, *i.e.*, of the moving part of the aerostatic linear bearing, *v*_1_, is measured as the Doppler shift frequency, *f*_Doppler1_, which can be expressed as follows:

(2) $$v_{1}=\lambda_{\text{air}}(f_{\text{Doppler}1})/2$$

(3) $$f_{\text{Doppler}2}=-(f_{\text{beat}1}-f_{\text{rest}})$$

where λ_air_ is the wavelength of the signal beam under the experimental conditions, and *f*_beat1_ is the beat frequency, which is the frequency difference between the signal beam and the reference beam and appears as the beat frequency at PD1. The position of the mass *x*_1_, the acceleration of the mass *a*_1_ and the force acting on the mass, *F*_mass_, are numerically calculated from the velocity.

On the other hand, the velocity of the sensing point of the force transducer, *v*_2_, is measured as the Doppler shift frequency, *f*_Doppler2_, which can be expressed as follows:

(4) $$v_{2}=\lambda_{\text{air}}(f_{\text{Doppler}2})/2$$

(5) $$f_{\text{Doppler}2}=-(f_{\text{beat}2}-f_{\text{rest}})$$

The beat frequency *f*_beat2_ is measured using PD2. The position *x*_2_ and the acceleration *a*_2_ of the actuator are numerically calculated from the velocity *v*_2_.

For simultaneous measurement, three frequency counters and the dynamic strain recorder (DSR) are triggered by a single signal originated by a light switch composed of a laser diode (LD) and a PD.

The force measured by the transducer, *F*_trans_, is calculated using the output signal of the transducer *V*_trans_ stored by a dynamic strain recorder (DSR; model: DC-204R, Tokyo Sokki Kenkyujo, Tokyo, Japan) and its static calibration result. The force calculated using the output signal of the transducer and its static calibration result, *F*_trans_ is compared with the force measured as the inertial force of the mass, *F*_mass_.

In the experiments, one set of experiment consists of 20 impact measurements. The first set of the experiments using Transducer-1, Set_A1, is conducted for evaluating the parameters for dynamic error correction. Then, additional three sets of experiments using all the three transducers are conducted for evaluating the validity of the estimated parameters. These additional three sets of the experiments using Transducer-1, Transducer-2 and Transducer-3, are described as Set_B1, Set_B2 and Set_B3, respectively.

## 3. Estimation of the Parameters for Dynamic Error Correction

**Figure 2.**
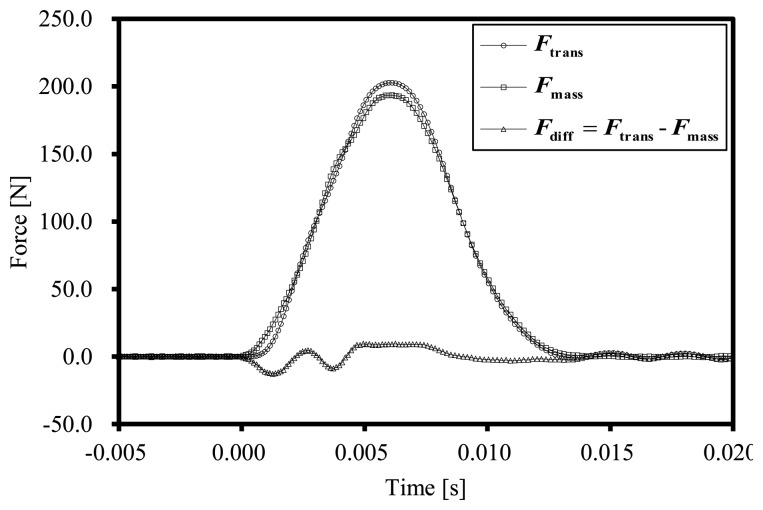
The force measured with the transducer *F*_trans_, the force measured with the interferometer *F*_mass_ and the difference between the forces.

Figure 2 shows the result of a single set of the impact tests of Transducer-1. Figure 2 shows the force measured by the force transducer *F*_trans_, and the force acting on the mass *F*_mass_ and their difference *F*_diff_:

(6) $$F_{\text{diff}}=F_{\text{trans}}-F_{\text{mass}}$$

**Figure 3.**
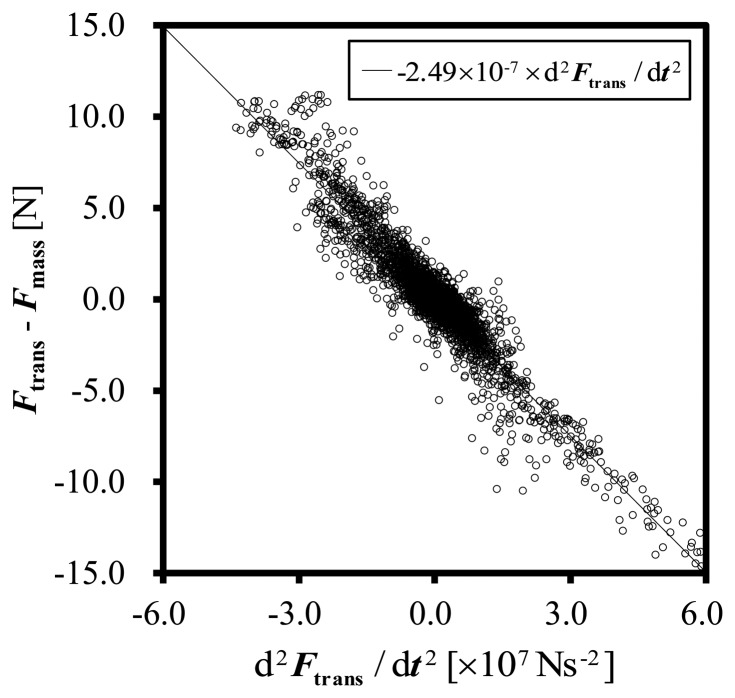
Relationship between the differential coefficient of the second order of the force measured with the transducer and the difference between *F*_trans_ and *F*_mass_.

The figure shows the electric response of the force transducer under the impact load. The difference between *F*_trans_ and *F*_mass_ is derived mainly from the difference between the static and dynamic characteristics of the transducer. The root mean square (RMS) value of the difference between *F*_trans_ and *F*_mass_ during the collision period, 0 ms < *t* < 14 ms, was approximately 6.2 N. Figure 3 shows the relationship between the second time derivative of the force measured using the force transducer, d^2^*F*_trans_/d*t*^2^, and *F*_diff_, for Set_A1.

The plots show a linear relationship between d^2^*F*_trans_/d*t*^2^ and *F*_diff_. The solid line in Figure 3 shows a regression line of the following equation.

(7) $$F_{\text{reg}}=C({\text{d}}^{2}F_{\text{trans}}/\text{d}t^{2})$$

The parameter for dynamic error estimation *C* is obtained as the slope of the regression line. Here, the regression line is determined by a least-square method and resulted in *C* = −2.49 × 10^−7^ s^2^. The measured plots coincide well with the regression line.

**Figure 4.**
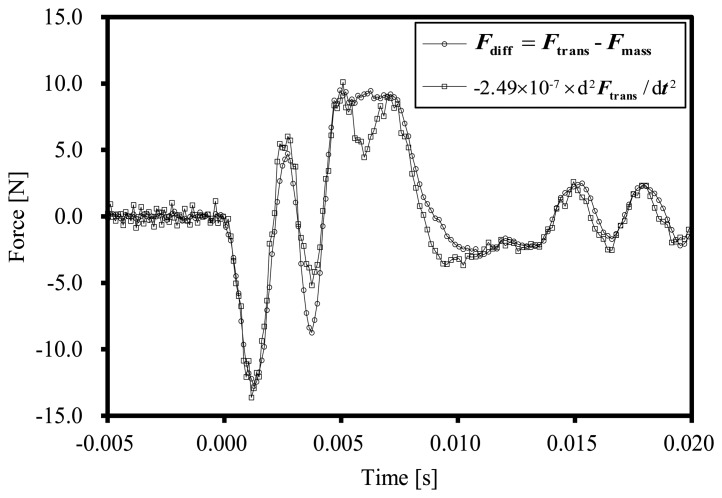
Difference between *F*_trans_ and *F*_mass_ and estimated inertial force of the transducer.

Figure 4 shows the difference between *F*_trans_ and *F*_mass_ and inertial forces of the transducer estimated with the parameter *C*, *F*_reg_. The measurement error, *F*_diff_, is almost the same as the result of the regression analysis, *F*_reg_. From this result, the corrected force, *F*_corrected_, can be calculated using following equation:

(8) $$F_{\text{corrected}}=F_{\text{trans}}-F_{\text{reg}}=F_{\text{trans}}-C({\text{d}}^{2}F_{\text{trans}}/\text{d}t^{2})=F_{\text{trans}}-(-2.49\times 10^{-7})({\text{d}}^{2}F_{\text{trans}}/\text{d}t^{2})$$

**Figure 5.**
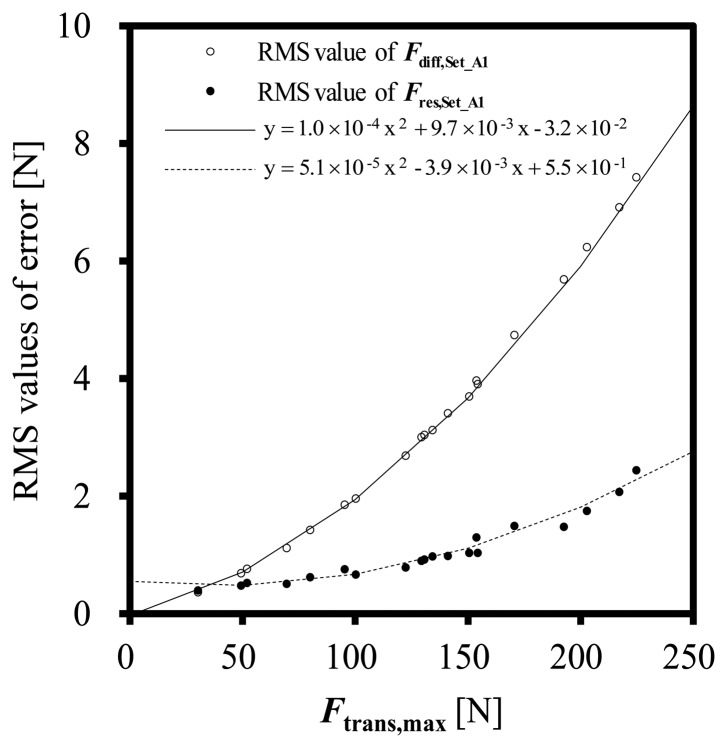
RMS valuses of the dynamic measurment error *F*_diff_ = *F*_trans_ − *F*_mass_ and RMS values of the residual error of the corrected values *F*_res_ = *F*_corrected_ − *F*_mass_.

Figure 5 shows RMS values of the dynamic errors *F*_diff_ and that of residual errors after dynamic correction *F*_res_:

(9) $$F_{\text{res}}=F_{\text{corrected}}-F_{\text{mass}}$$

for Set_A1. The RMS values of *F*_diff_ and *F*_res_ seem to be almost square functions of the maximum force *F*_trans,max_.

Solid and dashed curves in Figure 5 are regression curves of the RMS values of *F*_diff_ and *F*_res_, respectively. The RMS value of the differences between *F*_diff_ and its regression line and that between *F*_res_ and its regression line are 1.34 × 10^−1^ N and 3.58 × 10^−1^ N, respectively. This results show that the RMS values of the *F*_diff_ and *F*_res_ can be estimated from the *F*_trans,max_.

## 4. Evaluation of the Validity of the Estimated Parameters

**Figure 6.**
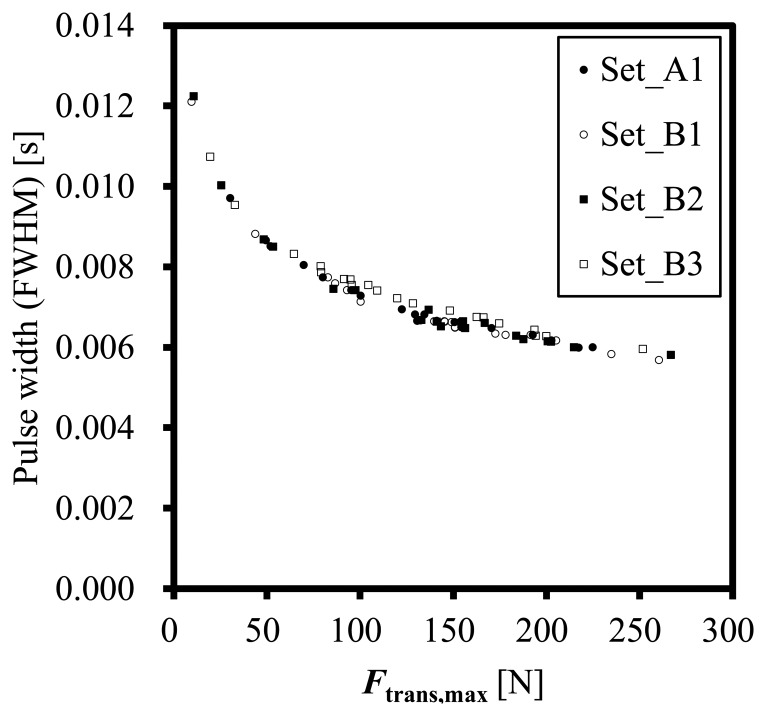
Pulse widths of *F*_trans_ against various maximum forces measured by using the three transducers.

To evaluate the validity of the dynamic error correction using the parameter *C* estimated using Transducer-1, the dynamic error correction is applied to the other sets of experiments performed by the three transducers, *i.e.*, Set_B1, Set_B2 and Set_B3. Figure 6 shows relations between the full width at half maximum (FWHM) of *F*_trans_ and the maximum force, *F*_trans,max_ for all the four sets of the experiments. All the sets show the similar relation.

**Figure 7.**
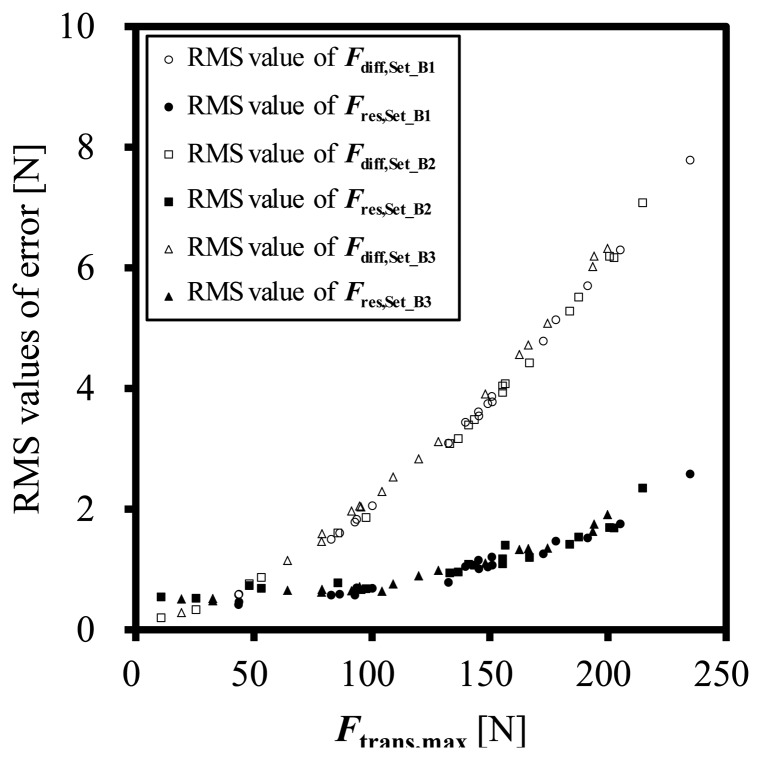
RMS values of *F*_diff_ = *F*_trans_ − *F*_mass_ and *F*_res_ = *F*_corrected_ − *F*_mass_ about other 20 sets of impact test with the three transducers.

Figure 7 shows the RMS values of the dynamic error *F*_diff_ and that of residual error after dynamic correction *F*_res_, about Set_B1, Set_B2 and Set_B3. The RMS values of *F*_res_ are effectively smaller than that of *F*_diff_. The correction with the parameter *C* has enough of a result even though Transducer-1 is 8 years older than the other two transducers.

## 5. Estimation of Uncertainty

The standard uncertainty *u*_trans_ in the un-corrected force *F*_trans_ is estimated as follows:

(10) $${\text{u}}_{\text{trans}}=\sqrt{{{\text{u}}_{\text{diff}}}^{2}+{{\text{u}}_{\text{LMM}}}^{2}}$$

where *u*_diff_ is the RMS value of *F*_diff_ during the impact shown in Figure 5. In the experiments, *u*_LMM_ is estimated to be 1.2 × 10^−4^ N[7] and is negligible. As shown in Figure 5, the *u*_diff_ can be assumed as a square function of the peak value of the force *F*_trans,max_ as follows:

(11) $${\text{u}}_{\text{trans}}={\text{E}}_{\text{S},1}\cdot {\text{F}}_{\text{trans},\text{max}}^{2}+{\text{E}}_{\text{S},2}\cdot {\text{F}}_{\text{trans},\text{max}}+{\text{E}}_{\text{S},3}$$

where the constants *E*_S,1_, *E*_S,2_ and *E*_S,3_ are parameters for estimating the dynamic error in the un-corrected force *F*_trans_ calculated based on the conventional static calibration.

As same as the *u*_trans_, the standard uncertainty *u*_corrected_ in the corrected force *F*_corrected_ is estimated as follows:

(12) $${\text{u}}_{\text{corrected}}=\sqrt{{{\text{u}}_{\text{res}}}^{2}+{{\text{u}}_{\text{LMM}}}^{2}}$$

where *u*_res_ is the RMS value of the residual error *F*_res_ during the impact shown in Figure 5. The *u*_res_ can be assumed as a square function of the peak value of the force *F*_trans,max_ as follows:

(13) $${\text{u}}_{\text{corrected}}={\text{E}}_{\text{D},1}\cdot {\text{F}}_{\text{trans},\text{max}}^{2}+{\text{E}}_{\text{D},2}\cdot {\text{F}}_{\text{trans},\text{max}}+{\text{E}}_{\text{D},3}$$

where the constants *E*_D,1_, *E*_D,2_ and *E*_D,3_ are parameters for estimating the residual error in the corrected force *F*_corrected_ estimated by means of the proposed dynamic correction method.

## 6. Correction Software

**Figure 8.**
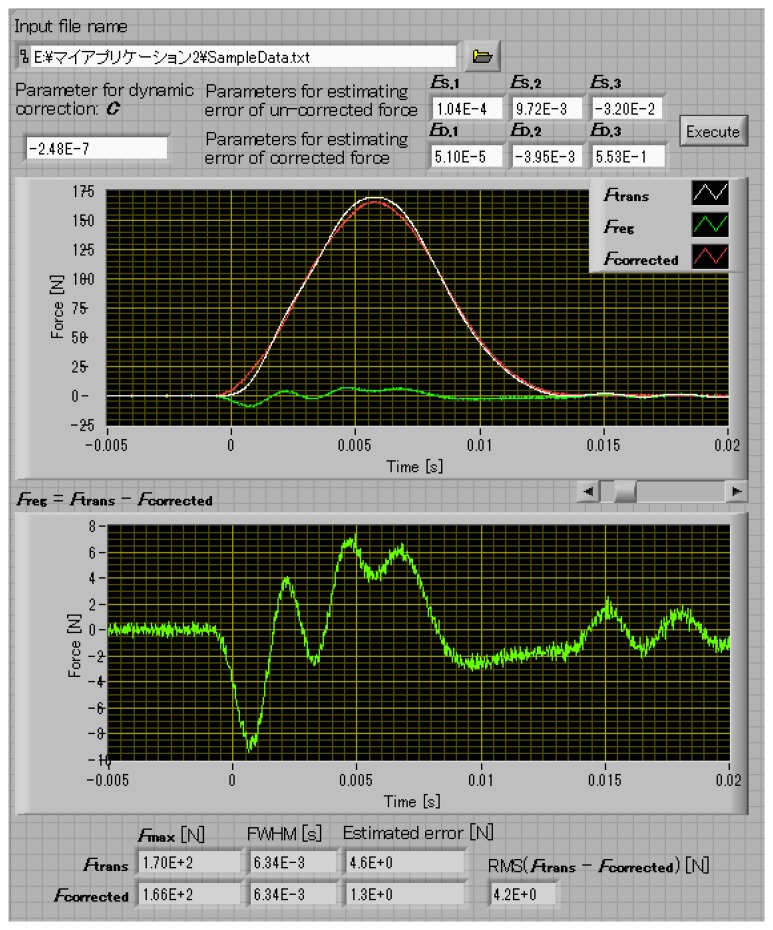
Screenshot of the correction software.

From the knowledge obtained from the above experiments, software that corrects the dynamic error in the impact force measurement is developed [8]. It works in the Microsoft Windows environment. Figure 8 shows a screenshot of the software.

This software processes the input waveform measured by the transducer based on the static calibration result, *F*_trans_, into (a) waveform of dynamic error *F*_reg_ [= *C*(d^2^*F*_trans_/d*t*^2^)]; (b) waveform of corrected force *F*_corrected_ (= *F*_trans_ − *F*_reg_); In addition, this software calculates: (c) the standard uncertainty *u*_trans_ in the un-corrected force *F*_trans_; and (d) the standard uncertainty *u*_corrected_ in the corrected force *F*_corrected_.

The software outputs them to a output file and shows them to the monitor window. The format of the input waveform file is a text format described the sampling interval in time and the sequence of force in newton calculated based on the static calibration result. The format of the output data file is a CSV format. The input data and the output data of the developed software are as follows,

(A) Input data, which should be manually input from the operational window.
  (1) Name of input text-file, in which the data set of the force calculated based on the static calibration results *F*_trans_(*i*) with the measurement time *t*(*i*) contains: [*t*(*i*), *F*_trans_(*i*)] (0 ≤ *i* < N − 1).
  (2) Parameter for dynamic error correction: *C*.
  (3) Parameters for estimating the dynamic error in the un-corrected force *F*_trans_ calculated based on the conventional static calibration: *E*_S,1_, *E*_S,2_ and *E*_S,3_.
  (4) Parameters for estimating the residual error in the corrected force *F*_corrected_ estimated by means of the proposed dynamic correction method: *E*_D,1_, *E*_D,2_ and *E*_D,3_.
(B) Output data, which is written in a CSV-file and shown on the display:
  (1) Waveform of *F*_trans_, *F*_reg_ [= *C*(d^2^*F*_trans_/d*t*^2^)] and *F*_corrected_ (= *F*_trans_ − *F*_reg_).
  (2) Parameters for *F*_trans_: the maximum value *F*_trans,max_, the pulse width FWHM_trans_ and estimated error *u*_trans_.
  (3) Parameters for *F*_corrected_: the maximum value *F*_corrected,max_, the pulse width FWHM_corrected_ and estimated error *u*_corrected_.
  (4) RMS value of the difference between *F*_trans_ and *F*_corrected_.

## 7. Conclusion

The validity of the proposed method for correcting the dynamic error in the impact force measurement is experimentally evaluated using three force transducers of the same model. Based on the experimental results, software, which corrects the dynamic error of force transducers using their own output signal, has been developed. Because there are no dynamic calibration methods for force transducers available at this moment, we believe that the work described in this paper is very valuable.

There are, however, still residual errors in the corrected force. For example, there is a small error factor which is assumed to result from the viscosity of the internal structure of the transducer. For more accurate correction and more accurate estimation of the uncertainty of measurement, our group is working on more accurate measurement of the force with the LMM and investigating the causes of the residual errors. Additionally, we will apply this correction method to transducers of other types and other models with wider ranges of forces in the future.

In this paper, the dynamic error correction is performed considering only with the inertial force of a part of the transducer itself. If a certain part is attached to the sensing point of the transducer and the force acting to the opposite side of the attached part should be measured, then the correction parameter should be modified for the mass of the attached part.

If the sensor manufacturers were to determine the correction parameters for each model using the proposed method; and provide the software with the parameters corresponding to each model; then the users could obtain waveforms corrected against dynamic errors and their uncertainty. We believe that in the near future; all the manufacturers of force transducers will provide similar software and the parameters corresponding to their products, so that transducer users will be able to perform dynamic error corrections and remove their uncertainty easily.
